# Spatio-temporal variations of strontium isotope ratios in the Mur River: a tool to support river management

**DOI:** 10.1007/s00027-025-01253-4

**Published:** 2026-01-17

**Authors:** Ulrike Moser, Barbara Čeplak, Stefan Wagner, Shaun T. Lancaster, Martin Šala, Thomas Prohaska, Gorazd Žibret, Johanna Irrgeher

**Affiliations:** 1 Department General, Analytical and Physical Chemistry, Chair of General and Analytical Chemistry, Franz Josef-Straße 18, 8700 Leoben, Austria; 2 Geological Survey of Slovenia, Dimičeva Ulica 14, 1000 Ljubljana, Slovenia; 3 National Institute of Chemistry, Hajdrihova Ulica 19, 1000 Ljubljana, Slovenia; 4 Department of Physics and Astronomy, University of Calgary, Calgary, T2N 1N4 Canada

**Keywords:** Environmental monitoring, Sr isotopes, Multi-element analysis, River management, River catchment tracing

## Abstract

This study explores the application of strontium (Sr) isotope ratios (^87^Sr/^86^Sr) as geochemical tracers to understand hydrological connectivity and anthropogenic influences in a geologically diverse Alpine river catchment encompassing regions of Austria and Slovenia. Focusing on the Mur River and its tributaries, water samples were collected across 45 sites during three hydrological regimes to capture seasonal and spatial variability. In total, 28 tributaries were sampled to evaluate their influence on the isotope composition of the main river. Isotope analyses using multi-collector inductively coupled plasma mass spectrometry, supplemented by geospatial mapping and multivariate statistics, were combined with measurements of 26 elemental concentrations to investigate land–water interactions and human impacts. The results demonstrate substantial temporal and spatial variability in ^87^Sr/^86^Sr ratios (ranging from 0.70853 to 0.71322), reflecting both geological heterogeneity and varying tributary inputs. A preliminary aquatic isoscape was developed, enabling the monitoring of connectivity and the impacts of geological and human influences under varying flow regimes. Tributaries, such as the Mürz River, significantly modulate isotope signatures in the main channel, often overriding local geological signals. The application of isotope pattern deconvolution revealed a contribution of 17% from the Mürz River to the Mur River’s isotope signature downstream of the confluence. Correlations between ^87^Sr/^86^Sr and metal concentrations further suggest the utility of ^87^Sr/^86^Sr as a proxy for source identification and anthropogenic influence assessment. By constructing a preliminary strontium isoscape for the region, this research provides novel insights into riverine connectivity, catchment-scale processes, and ecosystem dynamics. The integration of isotope data contributes to interdisciplinary understanding across geological, chemical, and ecological boundaries, and supports the development of isotope-based tools for sustainable aquatic system monitoring and management, including potential applications in fish ecology and habitat tracking.

**Graphical abstract:**

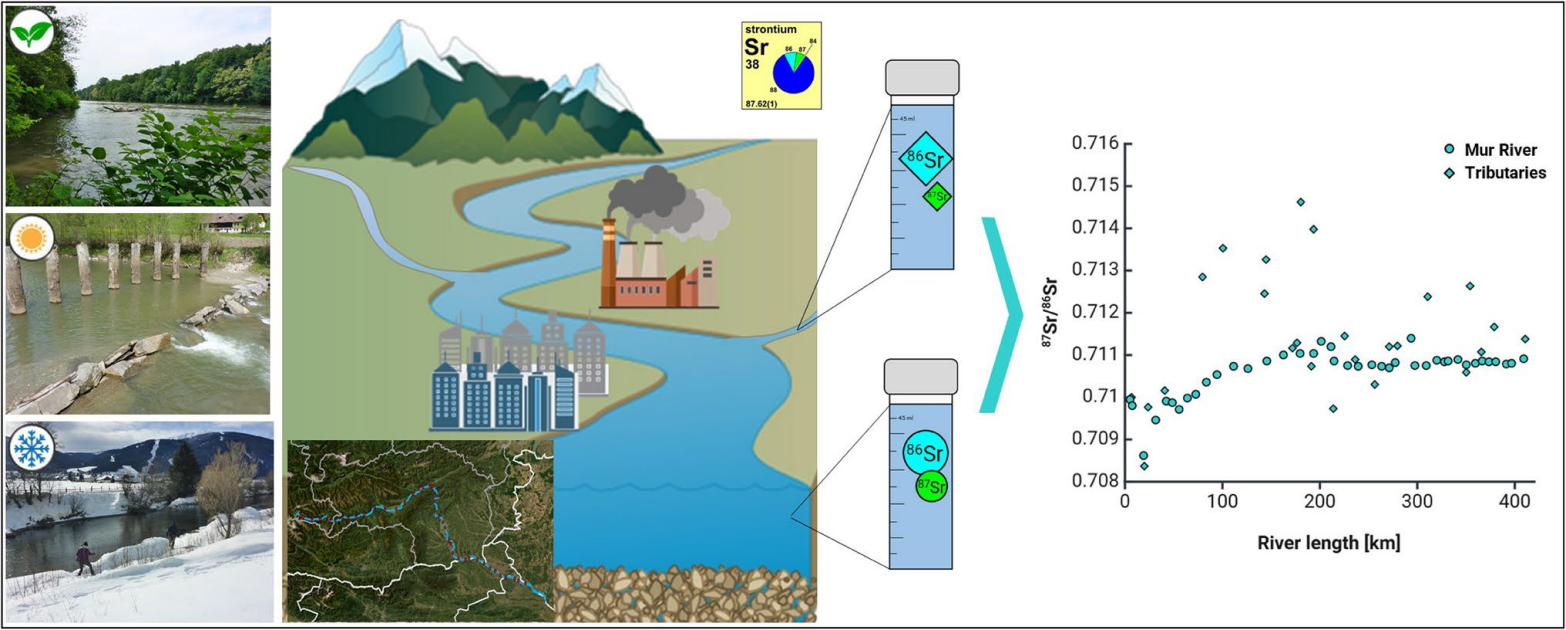

**Supplementary Information:**

The online version contains supplementary material available at 10.1007/s00027-025-01253-4.

## Introduction

Climate change has had a profound impact on ecosystems, particularly river landscapes, resulting in the loss of biodiversity and habitat alteration. Rivers such as the Colorado River (Miller et al. 2011) and the Amazon River (Sorribas et al. 2016) are experiencing reduced flows owing to rising temperatures and human over-extraction, threatening the flora and fauna dependent on these habitats (Poff et al. 1997). Over-management through damming and water diversion disrupts natural processes (Pringle 2000), while invasive species thrive in these changing environments (Mooney and Cleland 2001). The European Union is addressing these challenges with initiatives such as the Water Framework Directive, promoting re-naturalization to restore ecological balance and degraded ecosystems, while simultaneously enhancing climate resilience (European Commission 2020). Globally, effective management and international cooperation are crucial to sustain these vital ecosystems amid increasing environmental pressures (Biffi et al. 2021).

To enhance our understanding of ecological interconnectivities and biogeochemical processes, there is an increasing need for novel analytical tools that can provide insights into the interactions between various elements in the environment. The assessment of natural variations in the isotopic composition of the elements represents one such powerful tool, enabling the tracing of elemental sources, pathways, and transformation processes across complex environmental systems (Zimmermann et al. 2020; Holt et al. 2021; Sánchez‐Murillo et al. 2024). Radiogenic strontium (Sr) isotope amount ratios (*R* = *n*(^87^Sr)/*n*(^86^Sr)—further referred to as ^87^Sr/^86^Sr ratio) have been proven particularly effective in revealing connections, offering insights into sediment provenance, water movement, and nutrient processes (Bentley 2006; MacKenzie et al. 2019).

The half-life of ^87^Rb has been determined to be 49.61 ± 0.16 Ga, corresponding to a decay constant λ_87_ = (1.3972 ± 0.0045) × 10^–11^ a^−1^ (Villa et al. 2015). This extremely long decay rate supports the assumption of a stable ^87^Sr/^86^Sr isotope signature in weathered geological material, provided that the mixing ratios of contributing water sources remain constant within uncertainties (Négrel and Petelet-Giraud 2005; Zitek and Schmutz 2012; Pearce et al. 2015; Zieliński et al. 2016). As these isotopes primarily originate from the weathering of rocks and minerals (with varying rates according to the rock type) (Bain and Bacon 1994; Voss et al. 2014; Stevenson et al. 2018), they establish a clear link between the ^87^Sr/^86^Sr ratios in the underlying bedrock and the environmental catchment, solidifying their role as robust tracers of geological and hydrological processes (Capo et al. 1998; Faure and Mensing 2005).

In recent decades, the analysis of systematic variations in the isotope composition of Sr in the geosphere has been a widely applied method in the fields of geology and geochemistry, with ^87^Sr/^86^Sr isotope ratios emerging as valuable markers in freshwater systems, owing to their unique ability to reflect geological substrates and, to a lesser extent, monitor anthropogenic influences on water chemistry (Capo et al. 1998; Faure and Mensing 2005). Case studies on the spatial distribution of Sr isotopes in river systems have been conducted in various catchments, including the Danube (Zitek et al. 2015), the Rhine (Tricca et al. 2019), the Elbe (Reese et al. 2019), the Krka River (Šariri et al. 2022), the Tarim Basin in China (Wang et al. 2018), the Fraser River catchment in Canada (Voss et al. 2014), the Nagara River in Japan (Ida et al. 2025), and the Amazon Basin (de Almeida Mereles et al. 2025), highlighting the relevance and comparability of such investigations.

^87^Sr/^86^Sr isotope ratios exhibit a pattern of variation, reflecting the geological characteristics of the catchment area. As such, ^87^Sr/^86^Sr isotopes provide a more stable signature over time, making them particularly useful for understanding long-term geological and hydrological processes (Capo et al. 1998). However, anthropogenic activities, such as mining and agriculture, can introduce variations in ^87^Sr/^86^Sr isotope ratios, underscoring the need to track the difference between natural and human-induced changes in aquatic isoscapes (Frei et al. 2020; Zieliński et al. 2016).

When using ^87^Sr/^86^Sr isotopes as geochemical indicators in aqueous ecosystems, several challenges must be addressed. A primary challenge is the spatial and temporal variability of ^87^Sr/^86^Sr isotope ratios within riverine environments leading to uncertainties, particularly in heterogeneous areas with varying geology (McArthur et al. 2020). Rivers often receive inputs from diverse geological formations along their courses, resulting in complex mixing scenarios that complicate interpretation (Cai et al. 2020). Seasonal hydrological changes, such as variation in rainfall and runoff, may further alter the isotope composition of river waters, making it difficult to establish consistent baselines for comparison (Darling 2004). In addition, anthropogenic activities, such as agriculture and urban development, may introduce isotopically distinct sources of Sr into river systems. For example, fertilizers and wastewater may possess different ^87^Sr/^86^Sr isotope signatures compared with natural geological sources, potentially shifting the results and leading to misinterpretation of the data (Zieliński et al. 2016).

Isoscapes are spatial maps representing the variation of isotope ratios across a landscape (Bataille et al. 2020; Bowen 2010; West et al. 2010). While isoscapes of bioavailable ^87^Sr/^86^Sr isotope ratios have received increased interest over the last decades, with many different fields of application ranging from ecology (Crowley et al. 2017; Reich et al. 2021; Wooller et al. 2021) to food authentication (Lugli et al. 2022) and archaeology (Spies et al. 2025), aquatic isocapes have been less studied, likely due to the higher dynamics of the system and the higher difficulty of prediction using metadata (Brennan et al. 2016).

Aquatic isoscapes are a particularly effective tool to elucidate hydrogeochemical processes shaping freshwater ecosystems, enabling a nuanced understanding of water source origins, migration patterns, and the influence of environmental changes (Das et al. 2022; Remmer et al. 2020; Glibert et al. 2019). On the basis of the initial work of focusing on the natural variability of ^87^Sr/^86^Sr isotope ratios in rivers and lakes, driven by underlying bedrock composition and weathering rates, ^87^Sr/^86^Sr isotope ratios are nowadays used as natural tracers, linking water chemistry directly to its geographical source (Shand et al. 2009). Further developments have expanded the application of aquatic isoscapes to address ecological questions, such as tracking animal migrations and understanding nutrient cycling patterns in ecosystems (Hobson et al. 2010). The integration of ^87^Sr/^86^Sr isotope ratios into ecological studies highlights their potential to serve as a bridge between geochemical and biological processes, offering a holistic view of ecosystem dynamics. To further investigate the individual sources contributing to the mixed signature of a river, isotope pattern deconvolution (IPD) can be used as a very powerful data evaluation tool in isotope research. The relative contribution of the different sources of an element or its species in complex mixtures can be deduced in a single sample by analyzing their isotope signatures, followed by data reduction based on multiple linear regression. Furthermore, several studies emphasize the role of IPD in environmental studies, where it aids in tracing the sources of pollutants and understanding biogeochemical cycles (Komárek et al. 2022; Tchaikovsky et al. 2017; Irrgeher et al. 2014).

The presented study establishes the basis for an aquatic isoscape by determining the ^87^Sr/^86^Sr isotope composition of water in the Mur River in Austria and Slovenia. The Mur River is a key water source in Styria, together with the Drava and Danube River, as part of the so-called “European Amazon” (Umweltverband WWF Österreich 2024) and a prime case study for the interaction between rivers and their catchment areas. In the 1960s, it was heavily polluted by industrial and agricultural waste (Ertl et al. 1965), mirroring challenges seen in rivers such as the Rhône and Elbe (Reese et al. 2019; Voss et al. 2011), as well as the Colorado River (Miller et al. 2011) and the Amazon River (Sorribas et al. 2016). This led to stronger water protection laws in Austria during the 1980s and 1990s, aligned with EU directives (Wasserrechtsgesetz, 1959), resulting in ecosystem recovery and the return of many aquatic species (Kammel and Mebert 2011). Today, challenges such as flooding, biodiversity loss, and sediment erosion persist. Re-naturalization projects, such as in St. Michael (2011–2021) (Mayr, 2023), aim to restore natural river dynamics and improve habitats. Similar initiatives are planned along the AustrianSlovenian border to combat erosion and declining fish populations (Senfter et al. 2021).

Hydropower plants significantly disrupt river ecosystems, particularly affecting fish species and their reproduction (Pinter et al. 2024; Curtean-Bănăduc et al. 2019). In Europe, dams fragment rivers and alter natural flows, impacting habitat connectivity and sediment transport. Austria’s Mur River, with 22 hydropower facilities generating over 1.5 TWh annually (Verbund 2024), exemplifies these issues. Species such as the endangered Danube salmon are particularly affected by migration barriers and habitat loss (Fjeldstad et al. 2018). While fish ladders aim to assist migration, their effectiveness is limited owing to design and behavioral factors. Overall, they do not fully address habitat fragmentation. Monitoring fish migration using Sr isotope ratios offers insights into habitat use and movement patterns (Zitek et al. 2023).

Other European rivers, including the Danube and the Rhine, face similar challenges due to hydropower and its associated environmental concerns, including disturbed fish migration. In this context, the Danube sturgeon represents an especially vulnerable species, necessitating particular attention (Friedrich et al. 2019; Schneider 2010). The Mur River provides insights into balancing energy needs with ecological concerns, such as restoring connectivity through sophisticated fish ladders, dam removal, or adaptive management strategies that maintain seasonal flow variability. In order to estimate future measures regarding river management, it is first necessary to ascertain the state of the river.

This work therefore aims to provide a basic understanding of how rivers interact with their environment by identifying key indicators and parameters that exert influence over a river catchment. Up to 45 individual samples were obtained along the Mur River during three different flow regimes and seasons. In addition, up to 28 tributaries were sampled across two seasons to evaluate their role as potential markers of geogenic and anthropogenic inputs. This comprehensive sampling effort supported the development of a detailed ^87^Sr/^86^Sr isotope ratio tracer basemap for the Mur River catchment in Austria and Slovenia, offering a valuable tool for tracking environmental processes and connectivity within the river system.

## Materials and methods

### Reagents and laboratory conditions

Preparatory laboratory work and measurements were performed in an ISO class 8 clean room. Reagent grade type I water (*σ* = 0.055 µS cm^−1^) was obtained from a Milli-Q water purification system (MilliQ IQ 7000, Merck-Millipore, Darmstadt, DEU). Nitric acid (HNO_3_, *w* = 65%, p.a. grade; Carl Roth GmbH, Karlsruhe, Germany) was purified using a perfluoralkoxy-polymer (PFA) sub-boiling distillation system (Savillex DST- 4000, Savillex, Eden Prairie, USA). Plastic consumables were precleaned by soaking for 24 h in diluted HNO_3_ (*w* = 3%) followed by rinsing with reagent grade type I water before drying in a laminar flow hood.

The Inductively Coupled Plasma (ICP) multi-element standard solution VI (Multi VI, Merck Certipur, Darmstadt, DEU) was used as calibration stock standard for quantification (see details in ESI-1.1). The riverine water reference material SLRS-6 from the National Research Council Canada (NRC, Ottawa, CAN) was used as matrix-matched certified reference material during quantification of elemental mass concentrations and as quality control.

Certified reference material NIST SRM 987 (highly purified SrCO_3_; stock solution containing 18 µg g^−1^ Sr prepared in 5% HNO_3_ by dissolution of SRM 987 strontium carbonate, according to Irrgeher et al. (2013; National Institute of Standards and Technology, Gaithersburg, USA), was used as bracketing standard for Sr isotope ratio analyses. A Zirconium (Zr; Inorganic Ventures, Christiansburg, USA) elemental standard was spiked into the samples and the bracketing standards to internally correct for variations in instrumental isotope fractionation (IIF). The ICP single element standard for Sr (*w*(Sr) = 1000 µg g^−1^; Inorganic Ventures, Christiansburg, USA) was used as additional in-house quality control solution with a significantly lower ^87^Sr/^86^Sr isotope ratio than the NIST SRM 987 (details are provided in section “^87^Sr/^86^Sr isotope ratios”).

### Study area and sample collection

#### Study area

**Fig. 1:**
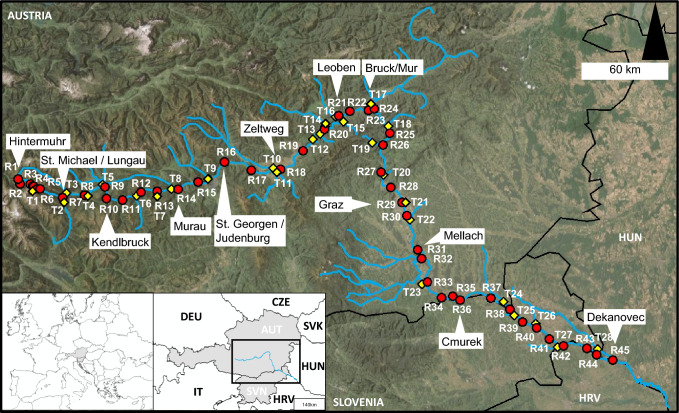
Sampling locations of the Mur River (“R” notations, red circles) and its tributaries (“T” notations, yellow diamonds). The main source area in Hintermuhr is highlighted, as are the cities with the highest populations: Graz, Leoben, and Bruck/Mur. In addition, the official water measuring stations used to source the information provided in Table 1 are highlighted. The last sampling point is located in Dekanovec at the Slovenian–Hungarian–Croatian border. Country codes follow ISO 3166–1

The Mur River (Fig. 1) has its origins in the Hohe Tauern National Park in the Hintermuhr valley, Salzburg, Austria. The river’s total length is 463 km. It flows through the Austrian federal state of Styria before forming a 34 km long border between Austria and Slovenia. Exiting Austria in Bad Radkersburg, the Mur River flows in a meandering course through a region of Slovenia considered ecologically important and is protected under Natura2000 (European Commission, 2025), a coherent network of protected areas within the European Union that has been established since 1992 in accordance with the provisions of the Fauna–Flora–Habitat Directive (The Council of the European Communities 1992). Here, the river forms the border between Slovenia and Hungary and Slovenia and Croatia until it converges with the Drava River in Croatia. The catchment area of the Mur River is recharged by groundwater and encompasses an expanse of over 14,000 km^2^, underscoring its significance in both regional hydrology and ecology (EEA 2014; Bundesministerium für Land- und Forstwirtschaft, Klima- und Umweltschutz, Regionen und Wasserwirtschaft, 2011).

On average, the Mur River experiences three different flow regimes during the course of a year. The period from April to June is characterized by elevated water levels because of snowmelt events. The period from July to October is dominated by mid water levels, complemented by single high water and flooding events due to precipitation from local thunderstorm activity. The period from November to March corresponds to periods of low water levels, as these seasons are characterized by comparatively low precipitation and significant snow accumulation, which retains water in the landscape (Land Steiermark 2024).

The Mur River catchment is further characterized by distinct geological sectioning. The uppermost section of the Mur River catchment, from the main source to Niederwölz, consists of high-grade metamorphic rocks of the Tauern window, such as schists, gneisses, and eclogites (Schmid et al. 2013). The mid-section, from St. Georgen ob Judenburg to Mellach, comprises a variety of metamorphic rocks originating from the Paleozoic era (Silvretta–Seckau–Nappe [Variscan]) and Drauzug–Gurk nappe system), sedimentary and volcanic deposits from the Permian and Mesozoic periods, and igneous intrusions (granites) of the Tertiary age (Gasser et al. 2009). The lowermost section of the study area in the Pannonian Basin “Grazer Palaeozoic” from Lebring to Decanovec, consists of Neogene to Quaternary clastic and carbonate sediments and outcrops of Pliocene alkali basaltic volcanic rocks (Kralj 2011; Flügel 1958).

#### Sampling

Water samples of the main course of the Mur River were collected during three main campaigns in May 2022, August 2022, and February 2023, which corresponded to the high, mid, and low water periods (Table 1). Owing to adverse weather conditions and subsequent challenges with accessibility to the source in the mountain plateau, the collection of a sample from the source of the Mur was conducted in August 2023. The sampling area spanned from the Mur River’s source in Salzburg, Austria, to the town Dekanovec located at the Slovenian–Croatian border, with the river being sampled at 40–41 sites, and distances between individual sites ranging from 7 to 13 km (Fig. 1), along with one tributary (T7 in Fig. 1). In June 2022, samples were taken from the upper course of the river as well as from ten tributaries, and in November 2023, samples were taken at 25 selected tributaries. Although the initial sampling design focused on the main river only, the tributaries were later identified as an area of additional interest during the course of the project. Consequently, tributary sampling was conducted in subsequent campaigns, subject to the availability of financial and logistical resources. The total number of water samples obtained for analysis across all sites resulted in 166 individual samples, with each sample being obtained in triplicates. The samples were collected at a depth of approximately 10 cm below the water surface, utilizing acid-cleaned vials for their storage. These samples were then stored at a temperature of 4 °C until further processing. On site, the pH, conductivity, and dissolved oxygen concentrations were measured using a portable multiparameter meter (Orion Star A329, Thermo Fischer Scientific, Waltham, USA). All sampling locations, individual samples, and a comprehensive dataset of the parameters are available in the Electronic Supplementary Information (ESI-2) in Tables ESI-2.1 to ESI-2.5.

**Table 1:** Water flow rates and water levels for selected locations on the date and time of the sampling. The data is provided by the governments of Salzburg and Styria (Land Steiermark 2024; Personal correspondence with Land Salzburg, Abt. 7/04 Hydrographischer Dienst, 9 January 2025)

Location	Season	Flow rate	Water level
		(m^3^ s^−1^)	(cm)
St.Michael/Lungau	May 2022	21.62	93.00
	Aug 2022	4.75	39.00
	Feb 2023	7.03	52.00
Kendlbruck	May 2022	57.35	152.00
	Aug 2022	17.54	96.00
	Feb 2023	8.36	78.00
Murau	May 2022	70.43	206.00
	Aug 2022	26.01	157.00
	Feb 2023	14.18	134.00
St. Georgen/Judenburg	May 2022	91.16	314.32
	Aug 2022	38.42	251.40
	Feb 2023	16.02	204.50
Zeltweg	May 2022	92.05	250.00
	Aug 2022	45.80	200.00
	Feb 2023	18.32	160.00
Bruck/Mur	May 2022	153.76	333.11
	Aug 2022	73.47	274.90
	Feb 2023	35.50	238.20
Graz	May 2022	150.46	408.00
	Aug 2022	62.08	346.00
	Feb 2023	30.40	293.00
Mellach/Wildon	May 2022	166.10	285.00
	Aug 2022	60.57	207.00
	Feb 2023	33.00	163.00
Cmurek	May 2022	209.18	301.02
	Aug 2022	76.50	231.11
	Feb 2023	64.65	232.73

### Sample preparation

#### Sample preparation for the determination of elemental mass concentrations

At each sampling location, collected water samples (*V* = 1 L) were immediately filtered using 0.22 µm mixed cellulose ester membranes (Merck Certipur, Darmstadt, Germany). To ensure stability, the samples were acidified to a pH < 2 using ultrapure HNO_3_ (*w* = 60%; Merck Certipur, Darmstadt, Germany) and stored at 4 °C.

#### Sample and standard preparation for Sr isotope ratio analysis

Three water sample replicates per location (each of *V* = 50 mL) were filtered using 0.45 µm hydrophilic teflon digiFILTERS (SCP Science, Baie d’Urfé, Quebec, Canada). In order to achieve stability, the samples were acidified with HNO_3_, resulting in a mass fraction to *w*(HNO_3_) = 1% using sub-boiled HNO_3_ (HNO_3_, *w* = 65%, p.a. grade; Carl Roth GmbH, Karlsruhe, Germany). The samples were analyzed using Inductively Coupled Plasma Tandem Mass Spectrometry (ICP-MS/MS) (NexION 5000, PerkinElmer, Waltham, USA) to determine the mass concentrations of Sr as the analyte of interest along with Ca and Rb as possible interfering elements in Sr isotope ratio measurements (see comprehensive details in Tables ESI-2.1 to ESI-2.5).

Analyte/matrix separation for Sr isotope ratio analysis was on the basis of an automated column chromatographic procedure using a 3-mL bed column filled with DGA Resin (50–100 μm particle size, TrisKem International, Bruz, France) and the prepFAST-MC system (Elemental Scientific, Omaha, USA) according to Zimmermann et al. (2020) and Retzmann et al. (2017). For this, the samples were first acidified to a uniform HNO_3_ concentration of 2 mol L^−1^, before being loaded onto the column. Matrix-adapted QC solutions (SRM 987 (*w*(Sr) = 50 ng g^−1^), *w*(Ca) = 10 µg g^−1^, and *w*(Rb) = 10 ng g^−1^ (Inorganic Ventures, Christiansburg, USA) in *c* = 2 mol L^−1^ HNO_3_, and procedural blanks were included in each separation run for quality control.

For multi-collector inductively coupled plasma mass spectrometry (MC-ICP-MS), all samples and standards were diluted volumetrically to achieve a final Sr mass fraction of either 20 ng g^−1^ or 50 ng g^−1^ in dilute HNO_3_ (*w* = 2%), depending on the daily sensitivity of the instrument. Sr mass fractions in samples and standards were matched within ± 10%, with the intention of performing standard-sample-bracketing (SSB). In addition, samples and standards were spiked with Zr at mass fractions of either 60 ng g^−1^ or 150 ng g^−1^ for additional correction of potential drift in IIF during the measurement (Retzmann et al. 2017; Horsky et al. 2016; Irrgeher et al. 2013).

### Analysis

#### Elemental mass concentrations

Elemental mass concentrations in the water samples were assessed at the National Institute of Chemistry (NIC), Slovenia, using a Varian 715-ES ICP Optical Emission Spectrometer (Agilent Technologies, Santa Clara, USA) for Na, Mg, Ca, and K, and an Agilent 7850 ICP-MS (Agilent Technologies), operated with helium as collision gas for B, Ti, V, Cr, Fe, Ni, Cu, Zn, As, Se, Rb, Sr, Zr, Mo, Cd, Ba, Pb, Th, and U. To check for instrumental drift, the calibration curve was measured before, in between, and at the end of the experiment. Quantification was carried out using external calibration with traceable standards, specifically Merck ICP standard solutions. Blank samples were processed in the same manner as the measured samples, using HNO_3_ and HCl, both of ultrapure grade. The system was rinsed with HNO_3_ (*w* = 2%) after each sample.

Elemental mass concentrations of Al, Mn, and Sr were determined at Montanuniversität Leoben (MUL), and determined using an ICP-MS/MS (NexION 5000) for samples that were measured prior to Sr separations, and an ICP-MS (NexION 2000, PerkinElmer) for samples that had undergone Sr separation to check recoveries. To correct for instrumental drift, internal normalization with Indium (In; Inorganic Ventures, Christiansburg, USA) was performed, and quantification was carried out using external calibration with traceable standards (see details in Table ESI-1.T1). To ensure the quality of the measurement, an in-house quality control standard (QC; Table ESI-1.T2) was analyzed after every eighth sample, as well as HNO_3_ blanks (*w* = 2%) to monitor possible carry-over effects. The system was rinsed after each sample with HNO_3_ (*w* = 3%). Details of instrumental set-ups can be found in Table ESI-1.T3.

#### ^87^Sr/^86^Sr isotope ratio measurements

Isotope ratio measurements were conducted using a Nu Plasma HR (NP048, Nu Instruments, Wrexham, UK) MC-ICP-MS connected to an ESI SC-4 DX autosampler (Elemental Scientific). A desolvation nebulization membrane unit (Aridus II, Cetac, Omaha, USA) equipped with a PFA nebulizer (Microflow ST Nebulizer, Elemental Scientific) was used for sample introduction. Bottled argon (Ar; 99.999% purity, Linde Gas GmbH, Stadl-Paura, Austria) was employed as the sweep and sample gas. Samples were analyzed in six blocks including ten cycles each at an integration time of 10 s. The rinsing time was 200 s and was conducted after each sample with HNO_3_ (*w* = 3%). The instrument parameters can be found in Table ESI-1.T4.

In our laboratory, we routinely measure ^87^Sr/^86^Sr isotope ratios in a standard-sample-bracketing (SSB) sequence, using solutions of Sr (NIST SRM 987) whose concentrations are matched to the Sr levels in the samples, which are diluted individually to concentration range within ± 10%. In addition, both the samples and the bracketing standard are spiked with Zr to allow for a possible additional correction of IIF occurring during MC-ICP-MS measurements as an internal inter-elemental correction (Horsky et al. 2016; Irrgeher et al. 2013).

### Data processing

#### Elemental mass concentrations

Raw intensities were both blank corrected as well as normalized to In as the internal standard. The limits of detection (LOD) and limits of quantification (LOQ) for each analyte were calculated by multiplying the standard deviation of the average of the 2% HNO_3_ blanks by 3 or 10, respectively. Data evaluation was performed using MassHunter version 5.1 (Agilent Technologies), Syngistix (v3.5, Perkin Elmer), and Microsoft 365 Excel version 2108 (Excel, Microsoft, Redmond, USA).

#### ^87^Sr/^86^Sr isotope ratios

In our laboratory, three distinct approaches are routinely employed for data evaluation: approach 1: internal intra-elemental correction (commonly referred to as the “conventional approach”) (Kazlagić et al. 2023), where the intensity (*int*) ratio *int*(^88^Sr)/*int*(^86^Sr) is measured in the sample and used for correcting the IIF of the Rb-corrected ratio *int*(^87^Sr)/*int*(^86^Sr). Approach 2: external intra-elemental correction (SSB) using a certified standard solution of the same element (Sr) (NIST SRM 987). Approach 3: combined internal and external inter-elemental correction using external standards (with Zr as an internal standard in an SSB with NIST SRM 987). Further details regarding the different approaches for data evaluation can be found in our previous studies (Retzmann et al. 2017; Horsky et al. 2016; Irrgeher et al. 2013, 2016).

In this study, the results obtained for ^87^Sr/^86^Sr using the three approaches were found to be equivalent within their combined uncertainties, indicating that any of the methods would be applicable. Given its suitability, approach 2 (SSB using NIST SRM 987) was selected as the preferred method. Accordingly, the subsequent paragraphs present only the methodological details and results derived from this approach.

Blank correction: For data evaluation of Sr isotope ratios, the Nu Instruments Calculation Editor (NICE, Nu Instruments) and Microsoft 365 Excel version 2108 (Excel, Microsoft) were used. As reported in Horsky et al. (2016) and Retzmann et al. (2017), analytical blank correction of isotope ratios was accomplished using the measure zeros method in the Nu Plasma software (Nu Plasma v1.4.2049, Nu Instruments) and subtracting the blank signal obtained from an HNO_3_ blank solution (*w* = 2%) from the samples and bracketing standard. A new blank measurement was made after a sequence of eight sample measurements. By this approach, any potential minor isobaric interferences of Kr present in the Ar gas were accounted for. In addition, a procedural blank (reagent grade type I water) underwent filtration and chromatographic matrix separation as detailed in the section “Sample and standard preparation for Sr isotope ratio analysis,” resulting in a total blank level of less than 0.2 ng of Sr.

Rb correction: Residual Rb, causing an isobaric interference of ^87^Rb^+^ on ^87^Sr^+^, was corrected mathematically by measuring ^85^Rb^+^ and using the natural abundance ratio of *n*(^87^Rb)/*n*(^85^Rb)_nat_ = 0.38571 (IUPAC/CIAAW) (CIAAW 2024; Meija et al. 2016), corrected for IIF using the fractionation factor calculated on the basis of of the ^88^Sr/^86^Sr ratio. The assumption of equal IIF for Rb and Sr was made to correct for the IIF of Rb by applying Russell’s model (Eqs. 1–2) (Russell et al. 1978).

First, the fractionation factor *f* used for Rb correction is determined using Eq. 1

1 $${f}=\mathrm{ln}\left({\left[\frac{n({}^{88}\mathrm{Sr})}{n({}^{86}\mathrm{Sr})}\right]}_{\mathrm{cert}}\cdot {\left(\frac{{\mathrm{int}(88)}_{\mathrm{spl}}}{{\mathrm{int}(86)}_{\mathrm{spl}}}\right)}^{-1}\right)\cdot {\left(\mathrm{ln}\left(\frac{M({}^{88}\mathrm{Sr})}{M({}^{86}\mathrm{Sr})}\right)\right)}^{-1}$$

using [*n*(^88^Sr)/*n*(^86^Sr)]_cert_ = 8.37861.

Second, *f* is applied to determine the intensity of ^87^Rb via the measured intensity total intensity measured at mass 85 (int(85)_spl_).

2 $${\mathrm{int}({}^{87}\mathrm{Rb})}_{\mathrm{spl}}=\left({\mathrm{int}\left(85\right)}_{\mathrm{spl}}\right)\cdot {\left[\frac{n({}^{87}\mathrm{Rb})}{n({}^{85}\mathrm{Rb})}\right]}_{\mathrm{nat}}/{\left(\frac{M({}^{85}\mathrm{Rb})}{M({}^{87}\mathrm{Rb})}\right)}^{f}$$

The obtained voltage corresponding to ^87^Rb will be subtracted from the total signal at *m*/*z* 87 (Eq. 3)

3 $${\mathrm{int}({}^{87}\mathrm{Sr})}_{\mathrm{spl}}={\mathrm{int}(87)}_{\mathrm{spl}}-{\mathrm{int}({}^{85}\mathrm{Rb})}_{\mathrm{spl}}$$

For SSB, samples and corresponding standards were introduced in the instrument as follows: SSB_1_ – sample – SSB_2_, where SSB_1_ and SSB_2_ refer to the bracketing standard NIST SRM 987. The fractionation factor of the ^87^Sr/^86^Sr ratio is then calculated following Eqs. 4 and 5.

4 $${f}_{\mathrm{SSB1,2}}=\mathrm{ln}\left(\frac{{\left(\frac{n({}^{87}\mathrm{Sr})}{n({}^{86}\mathrm{Sr})}\right)}_{\mathrm{cert}}}{{\left(\frac{n({}^{87}\mathrm{Sr})}{n({}^{86}\mathrm{Sr})}\right)}_{\mathrm{SSB}}}\right)/\mathrm{ln}\left(\frac{M\left({}^{87}\mathrm{Sr}\right)}{M\left({}^{86}\mathrm{Sr}\right)}\right)$$

5 $${f}_{\mathrm{SSB}}=\frac{{f}_{{SSB}_{1}}+{f}_{{SSB}_{2}}}{2}$$

Eventually, the measured ratio (int(^87^Sr)/int(^86^Sr)) in the sample is corrected via *f*_SSB_.

6 $${\left[\frac{n({}^{87}\mathrm{Sr})}{n({}^{86}\mathrm{Sr})}\right]}_{\mathrm{spl}}={\left[\frac{\mathrm{int}({}^{87}\mathrm{Sr})}{\mathrm{int}({}^{86}\mathrm{Sr})}\right]}\cdot {\left(\frac{M\left({}^{87}\mathrm{Sr}\right)}{M\left({}^{86}\mathrm{Sr}\right)}\right)}^{{f}_{SSB}}$$

Procedural standards of NIST SRM 987 and an in-house single element Sr standard (Inorganic Ventures), which underwent chromatographic matrix separation using the ESI prepFAST-MC, resulted in values of ^87^Sr/^86^Sr = 0.71030 ± 0.00031 (*U*, *k* = 2; *n* = 15) for NIST SRM 987 and ^87^Sr/^86^Sr = 0.70861 ± 0.00013 (*U*, *k* = 2; *n* = 6) for the Sr in-house standards, respectively. Replicate measurements of pure solutions of NIST SRM 987 across all isotope ratio measurements resulted in ^87^Sr/^86^Sr = 0.71034 ± 0.00007 (*U*, *k* = 2; *n* = 35), in accordance with the certified value.

This proves full control of our analytical protocol.

Calculation of relative differences between samples: Triplicate water samples from the high water season in May 2022 were analyzed to ensure the homogeneity and reproducibility of the samples and sampling approach (ESI-2.6, ESI-2.8). To evaluate the difference of obtained isotope ratios between two samples (here generalized as *R*_spl1_ and *R*_spl2_), it is standard practice to calculate capital delta values (*∆*; Coplen 2011). An adapted approach was applied in Eq. 7.

7 $$\Delta {({}^{87}\mathrm{Sr}/{}^{86}\mathrm{Sr})}_{\mathrm{spl}1,\mathrm{spl}2}=\frac{{R}_{\mathrm{spl}1}}{{R}_{\mathrm{spl}2}}-1$$

By multiplying by 1000 and expressing the values in ‰, this approach enables direct comparison of the relative differences in isotope ratios with their associated uncertainties, thereby providing a more robust basis for assessing statistical significance.

#### Statistics on elemental mass concentrations and ^87^Sr/^86^Sr isotope ratios

Pearson’s correlation coefficient was employed to analyze a dataset comprising of normally distributed variables (Benesty et al. 2009; Bonett and Wright 2000). The statistical analysis of the correlation coefficient was conducted using Excel according to J. Miller and Miller, (2018). Principal component analysis (PCA) was additionally conducted on the measured sample sets. The PCA was performed in R (R Core Team, 2023).

#### Isotope pattern deconvolution

Isotope pattern deconvolution (IPD) was applied to resolve overlapping isotope signals in mass spectrometric data. IPD uses expected isotope distributions based on natural abundances to mathematically separate mixed isotope patterns, allowing for more accurate identification and quantification. For this study, natural strontium isotopes were computed and included in the deconvolution algorithm. The ^84^Sr, ^86^Sr, ^87^Sr, and ^88^Sr isotopes, along with their natural abundances, were utilized to calculate endmembers for the mixing behavior of the Mur River and its tributary Mürz. The calculation of the percentage share of each source was conducted independently, without the consideration of the contribution volumes of the aforementioned rivers. The present study is based on multiple linear regression in a simple Excel LINEST function using four equations with two unknowns.

All isotope ratios ${R}_{\mathrm{corr}}^{i}$ (IIF-corrected isotope ratios of one isotope of an element relative to a common denominator) are measured and corrected for instrumental isotopic fractionation in a first step. The corresponding corrected isotope abundances ${A}_{\mathrm{spl}}^{i}$ (total isotope abundances of a single isotope in the sample) are calculated as follows in Eq. 8:

8 $${A}_{\mathrm{spl}}^{i}=\frac{{R}_{\mathrm{corr}}^{i}}{{\sum }_{i=1}^{n}{R}_{\mathrm{corr}}^{i}}$$

The molar fraction *x* of one source of Sr (*x*_source1_) in a mixture of two Sr sources [Sr (*x*_source1_) and Sr (*x*_source2_)] can be calculated using Eq. 9:

9 $${x}_{\mathrm{source}1}=\frac{{N}_{\mathrm{source}1}}{{(N}_{\mathrm{source}1}+ {N}_{\mathrm{source}2})}$$

Therefore, the molar amount of the individual contributors to the isotopic composition in the blend can be calculated by deconvolution of the measured isotope abundances and considering all input parameters from the two Sr sources following a system of equations resulting in a matrix model (Eq. 10):

10 $$\left[\begin{array}{c}{A}_{\mathrm{sample}}^{84}\\ {A}_{\mathrm{sample}}^{86}\\ {A}_{\mathrm{sample}}^{87}\\ {A}_{\mathrm{sample}}^{88}\end{array}\right]=\left[\begin{array}{cc}{A}_{{\mathrm{source}}_{1}}^{84}& {A}_{{\mathrm{source}}_{2}}^{84}\\ {A}_{{\mathrm{source}}_{1}}^{86}& {A}_{{\mathrm{source}}_{2}}^{86}\\ {A}_{{\mathrm{source}}_{1}}^{87}& {A}_{{\mathrm{source}}_{2}}^{87}\\ {A}_{{\mathrm{source}}_{1}}^{88}& {A}_{{\mathrm{source}}_{2}}^{88}\end{array}\right]\cdot \left[\begin{array}{c}{x}_{{\mathrm{source}}_{1}}\\ {x}_{{\mathrm{source}}_{2}}\end{array}\right]+\left[\begin{array}{c}{e}^{84}\\ {e}^{86}\\ {e}^{87}\\ {e}^{88}\end{array}\right]$$

As there are more parameters (isotopic abundances) than unknowns (molar amounts) in the equation system, an error vector is included. A more detailed description of the calculation procedures can be found in Tchaikovsky et al. 2017 and Irrgeher et al. 2014.

#### Preparation of an aquatic isoscape precursor

The isoscape precursor, a map displaying ^87^Sr/^86^Sr isotope ratios in the Mur River in a colored scheme, was created using ArcMap 10.8.1 © 2020 Esri (Environmental Systems Research Institute, Redlands, USA).

### Uncertainty calculation

For elemental mass concentrations obtained at MUL, combined uncertainties (*u*_c_) were calculated considering the precision of replicate sample measurements, the stated purity of the calibration standard, and the uncertainty of the slope of the calibration to determine the uncertainty in the calculated mass concentration of the analyte in a sample from the calibration (Eq. 11) (Ellison and William 2012), where ${s}_{y/x}$ is the residual standard deviation, *b*_1_ the slope, *N* the number of calibration points, *C*_cal_ the concentration of the sample according to the calibration, $\overline{C }$ is the average concentration of calibration standards and *C*_i_ the concentration of the individual calibration standards. A simplified Kragten spreadsheet approach was applied to calculate the combined expanded uncertainty (*U*, *k* = 2).

11 $$u\left(Cal\right)= \frac{{s}_{y/x}}{{b}_{1}}\sqrt{\frac{1}{N} +\frac{{({C}_{cal}-\overline{C })}^{2}}{\sum {({C}_{i}-\overline{C })}^{2}}}$$

For elemental mass concentrations obtained from the NIC, the measurement uncertainties are expressed as standard deviations of three repeated measurements and were below 5%. For further consideration, these uncertainties are expressed as expanded uncertainties *U* (*k* = 2) = 10% RSD.

For element concentration ratios the expanded uncertainty *U* was calculated using the standard deviations ($s$) across all ratios multiplied with the square root of the number of samples *n* (*U*, *k* = 2) = $s$• $\sqrt{n}$).

A combined uncertainty budget of Sr isotope ratios was considered by applying a Kragten spreadsheet approach (Horsky et al. 2016).

## Results

### Spatio-temporal variability of ^87^Sr/^86^Sr isotope ratios and Sr mass concentrations in the Mur River catchment

**Fig. 2:**
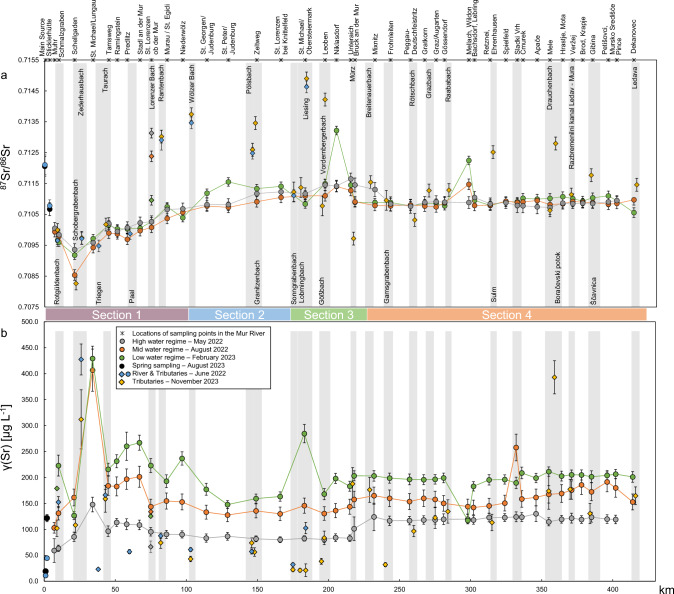
(**a**) ^87^Sr/^86^Sr isotope ratios from the Mur River and selected tributaries. The position of the tributary labels indicates if they tribute on the left side of the river in flow direction (top) or from the right side (bottom), with the exception of Schobergrabenbach, which also tributes the Mur River from the right side, and is marked at the top owing to space limitations. (**b**) Sr mass concentrations in µg L^−1^. Connecting lines between data points represent the main river. Error bars show expanded uncertainty *U* representing two SDs of replicate analysis of three individual samples

The source: In June 2022, a set of samples was collected in the very upper reaches of the Mur River. A sample taken approximately 10 m from the main source was found to have ^87^Sr/^86^Sr isotope ratios of 0.71211 ± 0.00032 [June 2022, sample R1 (source); Fig. 2a; ESI-2.3]. The sample from the source in August 2023 exhibited an ^87^Sr/^86^Sr isotope ratio of 0.71206 ± 0.00032 (August 2023, sample R1, ESI-2.3). The corresponding Sr mass concentrations were *γ* = 11.0 ± 0.9 µg L^−1^ (June 2022) and *γ* = 19.2 ± 0.4 µg L^−1^ (August 2023), respectively. Reference samples were collected approximately 3500 m downstream from the source at Sticklerhütte (Fig. 2a) in Hintermuhr. This was done on both occasions to establish a consistent baseline, allowing comparison with future source samples to determine whether isotope ratios remain stable despite changing external conditions. The reference samples exhibited ^87^Sr/^86^Sr isotope ratios of 0.71078 ± 0.00019 (June 2022) and 0.71067 ± 0.00021 (August 2023), and Sr mass concentrations of *γ* = 44.5 ± 3.5 µg L^−1^ (June 2022) and *γ* = 121.6 ± 4.7 µg L^−1^ (August 2023; Fig. 2b). Mur River samples R3–R5 (ESI-2.3), collected in June 2022, ^87^Sr/^86^Sr ranged from 0.71027 to 0.71048, along with Sr mass concentrations from *γ* = 94.0 ± 6.6 µg L^−1^ to *γ* = 144.3 ± 11.3 µg L^−1^.

**Fig. 3:**
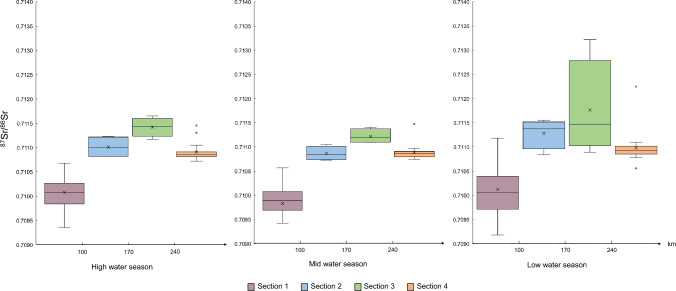
^87^Sr/^86^Sr isotope ratio distribution in the Mur River in respective sections in high (May 2022), mid (August 2022), and low (February 2023) flow regimes. The *x*-axis shows the distance in km of the Mur River from its source to the lower and upper bound of each section. The rectangular area (the “box”) shows the interquartile range (IQR), which represents the middle 50% of the data, between the first quartile (Q1, the 25th percentile) and the third quartile (Q3, the 75th percentile). The line within the box represents the median (Q2, the 50th percentile) of the dataset, indicating the central value. The whiskers represent the spread (minimum, maximum) of the data, excluding outliers. Data points outside of the whiskers are marked as individual points (circles) and represent outliers that are significantly higher or lower than the rest of the data. The mean values are indicated with an X

Sections of the Mur River: The riverine environment is characterized by isotope variations, which can be subdivided into four distinct subareas according to the Sr isotope ratio dynamics. From the main source until Niederwölz (Fig. 2a, sample R15), the isotope composition of the river is characterized by constant changes; however, the pattern of Sr isotopes remained stable across the seasons (section 1). The second zone (section 2), extending from Niederwölz to St. Lorenzen b. Knittelfeld (Fig. 2a, sample R19), is characterized by distinct seasonal variations, though less pronounced than that observed in the third zone (section 3), reaching further downstream to Frohnleiten (Fig. 2a, sample R26,). As can be seen in Fig. 3, the comparison of high, mid, and low water flow regime in section 3 provides a clear illustration of the seasonal fluctuations in the ^87^Sr/^86^Sr isotope signature, with distinctive peaks in Niklasdorf (^87^Sr/^86^Sr isotope ratios) and St. Michael/Obersteiermark (Sr mass concentrations). The subsequent course of the river to the Slovenian–Croatian border is characterized by the presence of a homogeneous ^87^Sr/^86^Sr isotope pattern, which persists throughout the river and across seasons, thereby establishing the fourth zone (section 4). However, an exception to this pattern is observed at the sampling point in Mellach, Wildon (sample R31), where a pronounced seasonal variation is evident. The ^87^Sr/^86^Sr isotope ratios in the river ranged from 0.70936 to 0.71165 in May 2022, 0.70853 to 0.71147 in August 2022, and 0.70918 to 0.71322 in February 2023 (Figs. 2a and 3; Tables ESI-2.1 to ESI-2.5; ESI-2.7). Looking at the geology of the four sections, section 1 is characterized by the high-grade metamorphic units of the Tauern Window, while sections 2 and 3 correspond to distinct segments of the Austroalpine nappes that differ substantially in geological composition. The boundary at Bruck/Mur is marked by the confluence with the Mürz tributary, which introduces material with distinct isotopic signatures. The transition between zones 2 and 3 reflects the increasing influence of left-bank tributaries draining Mesozoic nappes, characterized by higher ^87^Sr/^86^Sr ratios owing to their carbonate-rich lithologies, in contrast to the basement-dominated nappes of section 2. Therefore, it is expected that these left-bank tributaries have their distinctive ^87^Sr/^86^Sr signature (Fig. 2a).

**Fig. 4:**
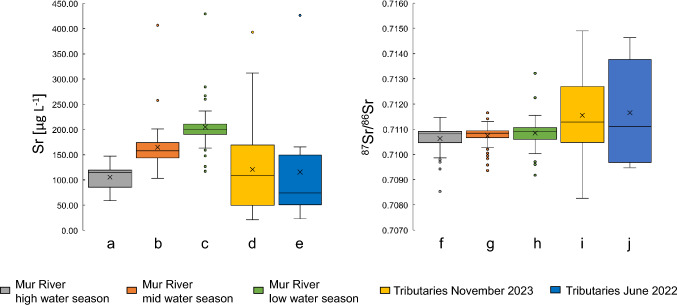
Distribution of the Sr mass concentrations and the ^87^Sr/^86^Sr isotope ratios of the distinctive seasons for the Mur River and its tributaries. (**a**–**c**) Distribution of Sr mass concentrations in the Mur River for high, mid, and low water regime from May 2022, August 2022, and February 2023, respectively. (**d**) The distribution of Sr mass concentrations in 25 tributaries obtained in November 2023. (**e**) Distribution of Sr mass concentrations of ten tributaries in the upper reaches of the Mur River in June 2022. (**f**–**h**) Distribution of ^87^Sr/^86^Sr isotope ratios in the Mur River for high, mid, and low water season in May 2022, August 2022, and February 2023, respectively. (**i**) Distribution of ^87^Sr/^86^Sr isotope ratios in 25 tributaries obtained in November 2023. (**j**) Distribution of ^87^Sr/^86^Sr isotope ratios of ten tributaries in the upper reaches of the Mur River in June 2022

In May and August 2022, the mass concentrations of Sr in the Mur River were lowest at the first sampling point in Muhr (sample R4) with *γ* = 59.2 ± 22.7 µg L^−1^ (May) and *γ* = 103.0 ± 10.3 µg L^−1^ (August). During the low water regime, the lowest Sr mass concentration was *γ* = 117.2 ± 11.7 µg L^−1^ in Mellach, Wildon (sample R31). In all seasons the highest mass concentrations were determined in St. Michael/Lungau (sample R8) reaching *γ* = 147.8 ± 14.1 µg L^−1^ in May 2022, *γ* = 406.7 ± 40.7 µg L^−1^ in August 2022, and *γ* = 429.1 ± 42.9 µg L^−1^ in February 2023 (Fig. 4, box plots a–c). In general, higher Sr mass concentrations over the course of the river were determined during the low water season in February 2023, whereas the lower mass concentrations were found in the samples from the high water season in May 2022. This correlates with the water level and water discharges in their respective seasons (Table 1), with a Pearson’s correlation factor of > 91% for the upper part of the river (upstream Graz, sample R29) and 65–68% for Mellach and Cmurek (sample R36).

In a sampling campaign conducted in June 2022, ten selected tributaries were sampled and analyzed (Fig. 2; Table ESI-2.3). The ^87^Sr/^86^Sr values range from 0.70948 ± 0.00019 in the Triegen River (sample T4) to 0.71463 ± 0.00019 in the Liesing River (sample T14). The lowest Sr mass concentrations were observed in the Triegen River, with a value of *γ* = 23.1 ± 2.1 µg L^−1^, while the highest were found in Zederhausbach creek (sample T3) with a value of *γ* = 426.4 ± 29.9 µg L^−1^.

In November 2023, 25 tributaries (Fig. 2a; ESI-2.5) were selected on the basis of preliminary investigations, which indicated a potential for major impacts on the river. The ^87^Sr/^86^Sr isotope ratios ranged from 0.70826 ± 0.00021 in Schobergrabenbach (sample T2), in the upper region of the Mur River, to 0.71490 ± 0.00021 in the Liesing River (sample T14) in St. Michael/Obersteiermark. The Sr mass concentrations were the lowest in Lobmingbach *γ* = 21.2 ± 2.1 µg L^−1^ (sample T13), in St. Stefan/Leoben, and the highest in Boračevski potok *γ* = 393.0 ± 39.3 µg L^−1^ (sample T25) in Radenci. The distribution of the Sr mass concentrations and ^87^Sr/^86^Sr isotope ratios per season can be found in Fig. 4. Data for the boxplots are provided in ESI-2.6.

### Elemental mass concentrations, ratios, and method validation

The elemental mass concentrations of B, Na, Mg, Al, K, Ca, Ti, V, Cr, Mn, Fe, Ni, Cu, Zn, As, Se, Rb, Sr, Mo, Zr, Cd, Ba, Pb, and U can be found in ESI-2.1 to ESI-2.5. The results are further discussed in Chapter "Temporal variations of major elements and ^87^Sr/^86^Sr isotope ratios across three seasons".

The Sr mass concentration was normalized to the Ca mass concentration to allow comparison of Sr mass concentrations across seasons and to assess the potential impact of tributaries on the river. Fluctuations in Sr mass concentration (Fig. 2b) at the respective locations often corresponded to variations and decreases in the *γ*(Sr)/*γ*(Ca) ratio (ESI-1.3; Supplementary Fig. 1). Further results can be found in ESI-1.3 and ESI-2.1 to 2.5.

Results from method validation can be found in the method section in "^87^Sr/^86^Sr isotope ratios". Compiled data for triplicate analysis of samples can be found in ESI-1.4 as well as in ESI-2.8. The complete data set of all analyzed parameters in real samples along with sampling locations are provided in ESI-2.1 to ESI-2.9.

## Discussion

### ^87^Sr/^86^Sr isotope ratio dynamics in the Mur River

#### Geo-isotope sectioning of the Mur River

**Fig. 5:**
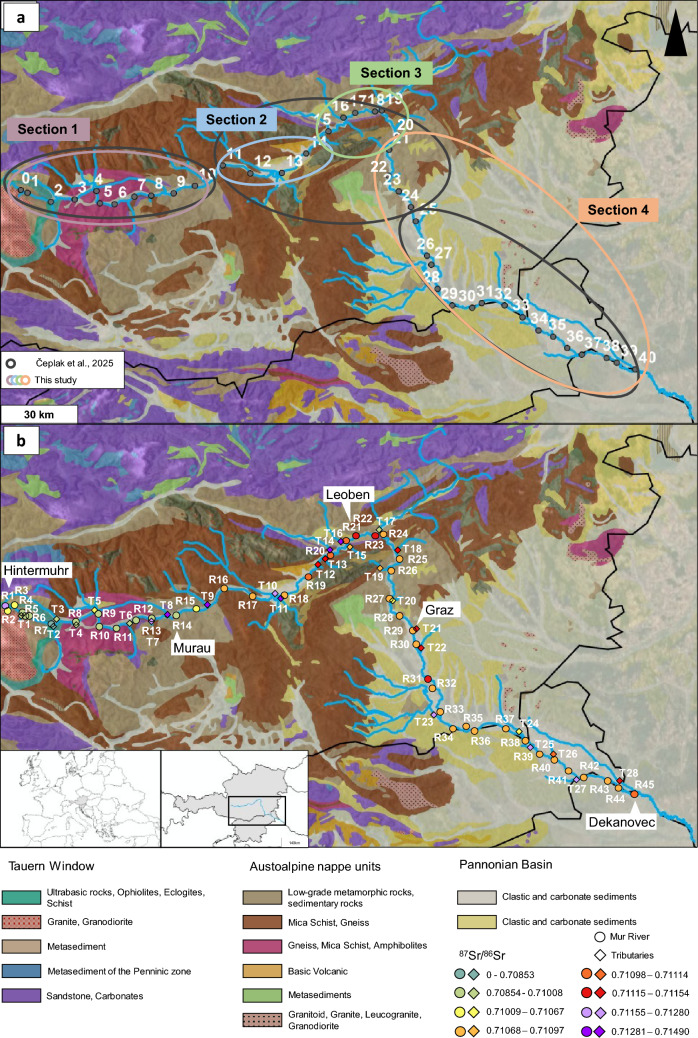
(**a**) Geological map of the sampling area provided by One Geology Europe, showing the differences in the division of the area by Čeplak et al. 2025 and this study. Notations, displaying sampling locations, according to Čeplak et al. 2025. (**b**) Aquatic basemap isoscape of ^87^Sr/^86^Sr isotope ratios in the Mur River (R) in June and August 2022 and August 2023, and its tributaries (T) in June 2022 and November 2023, embedded in a geological map of the Mur River catchment area in Austria and Slovenia

In Čeplak et al. (2025) the study area was divided into three sections (Fig. 5a), according to the geological background (see detailed description in “Study area”), on the basis of findings from stream and alluvial sediment samples. In the present study, the study area was subdivided in a similar way, with modifications reflecting the observations obtained from data obtained in the water phase. While the uppermost section of the river (section 1), from the main source to Niederwölz (R15, sample 10 in Fig. 5a) showed the lowest ^87^Sr/^86^Sr ratios (Fig. 2a) along the river, and is therefore in accordance with the suggested classification. Section 2 would, according to the ^87^Sr/^86^Sr data, be reduced in area (Fig. 5a, samples 11–14; Fig. 5b, samples R16–R19) and was defined as a transition section, with significant impacts from tributaries (samples T10 and T11). In the subsequent section (section 3; Fig. 5a, samples 15–19; Fig. 5b, samples R20–R24), seasonal fluctuations were identified. As the Mur River enters the sedimentary basin, its isotope composition remains fairly stable, which is in accordance with the geological background in section 4.

#### Spatial variations along the Mur River and tributary contributions

The upper reaches of the river exhibit multiple geological settings, such as Variscan basement rocks with quartz-sulfide veins as well as metamorphic sequences of Permian to Mesozoic cover, e.g., marble (Čeplak et al. 2025; Schmid et al. 2013; Horner et al. 1997). An increase of the ^87^Sr/^86^Sr isotope ratio is observed after Schellgaden (R7; Fig. 2a). In the area, Au-ore deposits in marble host rock are present. Also, the tributary Schobergrabenbach, which is a runoff from ancient Au-ore deposits (T2, ^87^Sr/^86^Sr = 0.70826 ± 0.00021) (Zieliński et al. 2016; Horner et al. 1997) may cause the change in the ^87^Sr/^86^Sr isotope ratio. While metamorphosis of carbonates (with known low ^87^Sr/^86^Sr isotope ratios (see “Influence of tributaries in sections 3 and 4, south of Bruck an der Mur”) to marbles can alter the Sr isotope signature, it can be assumed that the conditions of the metamorphosis have not affected the Sr isotope ratio significantly, hence explaining the ^87^Sr/^86^Sr isotope ratios in the tributary Schobergrabenbach, which corresponds best with the Sr isotope composition of seawater in the Cambrian period (Veizer et al. 1999). However, it must be noted, that deducing rock age by the isotope ratio in discharged water is not an established method for determining the age of rock. Thus, the latter finding has to be regarded as an indicator only. The Sr mass concentrations in St. Michael/Lungau (R3) can be attributed to the high mass concentration (*γ*(Sr) = 426.34 ± 29.6 µg L^−1^ in June 2022, ESI-2.3; *γ*(Sr) = 311.9 ± 31.2 µg L^−1^ in November 2023, ESI-2.4) observed in the tributary Zederhausbach (T3, Fig. 2b) that flows into the river before St. Michael/Lungau. Although it originates from a carbonate catchment area, which might be indicative of the high Sr mass concentrations, the Zederhausbach flows mainly though a similar geological setting as the Mur River catchment, which has undergone a transition from siliciclastic to carbonates (Fig. 5a).

As demonstrated in Fig. 2a, the creeks Zederhausbach (the relative isotopic difference expressed as *Δ*_spl1,spl2_ value between June 2022 and November 2023 is 0.02‰), Triegen (T4), Taurach (T5), and Paal (T6) show a similar isotope pattern to that of the main river, and derive from the same geological main setting (Fig. 5a, b). In the upper section of the study area (Table 1), the water discharge of the Mur River is approximately twice that of its tributaries, such as the Zederhausbach, with water discharges of 10.52 m^3^ s^−1^ during high water season, 2.03 m^3^ s^−1^ in mid water season, and 1.77 m^3^ s^−1^ in low water season. The substantial contributions of the said tributaries to the water body of the Mur River imply a considerable impact on its ^87^Sr/^86^Sr ratio composition.

The ^87^Sr/^86^Sr isotope ratios of the water samples from the tributaries Lorenzer Bach creek (T7), Rantenbach creek (T8), and the Wölzer Bach creek (T9) were in general higher than that of the Mur River. The aforementioned tributaries, as well as others with elevated Sr isotope ratios such as Pölsbach (T10), Granitzenbach (T11), Liesing (T14), and Vordernberger Bach (T16), are derived from the Silvretta–Seckau–Nappe system (see section “Study area”, Gasser et al. 2009), which, owing to its geology (old felsic), had sufficient time to decay ^87^Rb to ^87^Sr, resulting in a higher ^87^Sr/^86^Sr isotope ratio (Faure and Mensing 2005). The tributaries Sonngrabenbach (T12), Lobmingbach (T13), and Gößbach (T15) show similar Sr isotope ratios as the Mur River. Those tributaries derive from the Kraubath dunite-harzburgite massif, a strongly deformed and metamorphosed ophiolite sequence (Malitch et al. 2003).

The elevated Sr isotope ratios result in an influence on the Mur River throughout the seasons. The Lorenzer Bach creek contributes to the Mur River right after the sampling point at St. Lorenzen/Mur (R13). This tributary was sampled during the seasonal experiments of the Mur in May and August 2022 and in February 2023 to monitor seasonal effects on tributaries. It exhibits seasonal variations in its ^87^Sr/^86^Sr isotope ratios (0.71313 ± 0.00016 in May 2022, 0.71238 ± 0.00017 in August 2022, and 0.71096 ± 0.00016 in February 2023), which, when combined with the Rantenbach creek and its higher ^87^Sr/^86^Sr isotope ratio, can be directly converted to the Mur River visibly at the subsequent sampling point in Murau (R14), and results in an increase of the ^87^Sr/^86^Sr isotope ratio of the Mur River at this location.

Cross-seasonal stability is primarily controlled by the water discharges of the Wölzer Bach Creek and the Mur River at Murau (R14, Fig. 2). The water discharges for the river in Murau, as presented in Table 1, were compared with the water discharges for the Wölzer Bach creek (9 m^3^ s^−1^ in May 2022, 2.4 m^3^ s^−1^ in August 2022, and 1.42 m^3^ s^−1^ in February 2023). On the same day and at approximately the same time, the water discharges of Wölzer Bach exhibited a constant difference in all three seasons by a factor of eight to ten from the water discharges of the Mur. Along with the relative differences between ^87^Sr/^86^Sr isotope ratios in June 2022 and November 2023 of certain tributaries, reaching relative differences of 0.02–0.34‰, temporal stability could be shown. Hence, the cross-seasonal change in the isotope composition of the Mur River can be attributed to tributaries and their respective geological settings (Fig. 5b).

Figure 5b demonstrates a precursor for an aquatic isoscape, plotted on a geological map. The data presented in this study, derived from the Mur River, includes Sr isotope ratios from August 2022 (main river), August 2023 (main source), and November 2023 (tributaries). Additional sampling points from June 2022, which were not included in the August 2022 (main river) or November 2023 (tributaries) datasets, are shown on the map (data provided in ESI-2.1, ESI-2.3, and ESI-2.5). The August 2022 data was used for the main river owing to its greater comparability with the values obtained from the main source in August 2023. In addition, the August 2022 dataset is the most complete, as accessibility constraints limited sampling in May 2022 (R45) and February 2023 (R1). Single data points from June 2022 were included on the map for reference.

#### Tributary Rotgüldenbach: a closer look

**Fig. 6:**
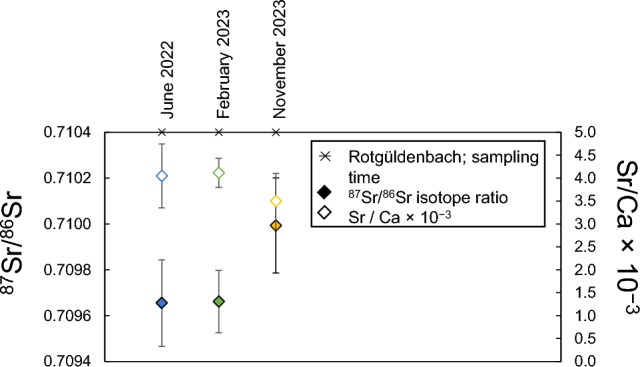
The ^87^Sr/^86^Sr isotope ratios, displayed in filled diamond symbols, of the creek Rotgüldenbach, a tributary of the Mur River, along with the *γ*(Ca) normalized Sr mass concentrations × 10^−3^ *U*(*k* = 2), *γ*(Sr)/*γ*(Ca), shown in empty diamond symbols. Error bars represent expanded uncertainties *U*(*k* = 2) (see "Uncertainty calculation")

To obtain a comprehensive overview of the seasonal variability of the tributary Rotgüldenbach (T1), three samples were collected in June 2022, February 2023, and November 2023. The area’s history as a mining deposit has led to an elevated As mass concentration in the Rotgüldenbach (ESI-2.3 to ESI-2.5), possibly influencing the Mur River behind the confluence (Wen et al. 2018; Horner et al. 1997). The values for the ^87^Sr/^86^Sr isotope ratios ranged from 0.70965 ± 0.00019 to 0.70999 ± 0.00021 (Fig. 6), the *γ*(As) ranged from 18.2 ± 2.0 µg L^−1^ to 39.6 ± 0.3 µg L^−1^ (see ESI-2.3 to ESI-2.5). A Pearson’s test revealed no correlation between *γ*(As) and ^87^Sr/^86^Sr isotope ratios, with a correlation factor of 0.015 and a *p*-value of 0.5.

An anticorrelation between Sr isotope ratios and water discharge was observed. The Sr mass concentrations in the Rotgüldenbach ranged from 186.5 ± 10.0 µg L^−1^ in February 2023 to 128.8 ± 15.7 µg L^−1^ in November 2023. To account for the impact of the creek discharge, *γ*(Sr) was normalized to *γ*(Ca) and is presented as the normalized *γ*(Sr)/*γ*(Ca) × 10^3^ ratio in Fig. 6. The Sr isotope ratios and discharge show a strong anticorrelation, with a Pearson correlation coefficient of −0.99 (*p*-value = 0.04), suggesting that the ^87^Sr/^86^Sr isotope ratio increases as discharge decreases. This phenomenon may be attributable to the impact of groundwater and precipitation, a subject that is further discussed in Chapter "Spatial variations along the Mur River and tributary contributions". To summarize, ^87^Sr/^86^Sr from the Rotgüldenbach and the Mur River do not differ significantly over seasons.

#### Influence of tributaries in sections 3 and 4, south of Bruck an der Mur

The tributaries Liesing (T14) and Vordernberger Bach (T16), which exhibit the highest ^87^Sr/^86^Sr ratios, both drain from Mesozoic (Late Triassic) units of the Austroalpine nappes, which are rich in carbonate rocks, such as those of the Dachstein formation (Richoz 2015), in contrast to the more silicate-dominated basement nappes of the central Austroalpine domain. The ^87^Sr/^86^Sr isotope composition and Sr mass concentrations of the Mur River have been observed to remain consistent after Bruck/Mur as the course of the Mur River turns to the south, coinciding with a transition in geology to quaternary sediments, with some exceptions. Of particular interest is the observation that during periods of high water levels, the tributary Mürz (^87^Sr/^86^Sr = 0.70972 ± 0.00021; T17) appears to exert a less significant influence on the main river as compared with low water level scenarios. An increase of the water discharge of the Mur River may be a contributing factor to this effect. The lower ^87^Sr/^86^Sr isotope ratio observed in the Mürz River can be explained by the fact that its source region is located in the Northern Calcareous Alps in Austria. These mountains are formed from seawater, which has a significantly lower Sr isotope signature (Spöt and Pak 1996). Further downstream, in Frohnleiten (R26), the ^87^Sr/^86^Sr signature of the Mur River remains very stable along the river and within seasons, and no significant influences of the tributaries Gamsgrabenbach, Rötschbach, Grazbach, and Raababach (T19–T22) were observed. This is likely because of the lower water discharges of these tributaries, the Mur River’s average discharge of 110 m^3^ s^−1^, its entry into the Graz sedimentary basin, and the similar ^87^Sr/^86^Sr isotope ratios between the main river and its tributaries. These tributaries flow through a predominantly similar type of host rock to that of the Mur River (Fig. 5b) (Zieliński et al. 2016). The isotope compositions of the tributaries Sulm (T23), Boračevski potok (T25), and Ščavnica (T27) exhibit distinctive Sr isotope patterns (Fig. 2b; ESI-2.5), and despite exhibiting high water discharges and water levels on their own, their water discharge is insufficient to result in a change of the isotope signature in the Mur River. Gas bubble formation was observed during sampling from the Boračevski potok creek, likely originating from fissures in the bedrock, as the area is known for elevated thermal activity (Kralj 2004). These activities led to higher mass concentrations of *γ*(Sr) = 393.0 ± 39.3 µg L^−1^ and *γ*(Fe) = 300.9 ± 30.1 µg L^−1^. The tributaries Drauchenbach (T24) and Razbremenilni kanal Ledav – Mura (T26) have been observed to display highly comparable ^87^Sr/^86^Sr isotope ratios to those observed in the Mur River. The last sampling point of the Mur River in Dekanovec (R45) was inaccessible in May 2022 owing to flooding. However, the slight distinctive isotope composition of the tributary Ledava (T28; ^87^Sr/^86^Sr = 0.71146 ± 0.00021) suggests the possibility of an influence on the main river, particularly given the observed variation in isotope composition in Dekanovec (^87^Sr/^86^Sr = 0.71097 ± 0.00017 in August 2022; Fig. 5b; ^87^Sr/^86^Sr = 0.71056 ± 0.00016 in February 2023). Thermal waters in the Styrian Basin appear not only in the Boračevski potok but also affect the Sulm, Ščavnica, and Ledava tributaries, likely contributing to shifts in their ^87^Sr/^86^Sr isotope signatures (Négrel and Petelet-Giraud 2005; Kralj 2004; Lenkey et al. 2002). A targeted ^87^Sr/^86^Sr analysis of these thermal waters would provide valuable insights into their role in modifying the isotopic composition of both the tributaries and the Mur River.

#### Temporal variations of major elements and ^87^Sr/^86^Sr isotope ratios across three seasons

**Fig. 7:**
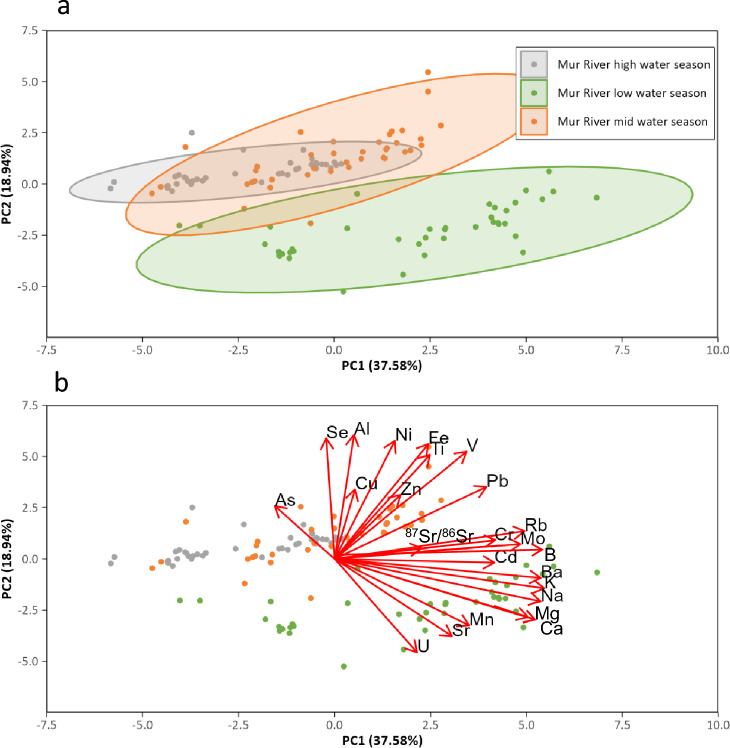
(**a**) The principal component analysis (PCA) plot is indicative of discrepancies in the major elements and Sr isotope ratios during the periods of high and low water regime. The mid water season plot extends over both the high and low water seasons, and appears to be influenced by components of the respective seasons. (**b**) The analysis reveals the loadings of the PCA and elucidates the influence of the major elements and Sr isotope ratios on the designated seasons

The application of PCA to datasets reveals the presence of two distinct seasonal clusters based on principal component 1 (PC1) and principal component 2 (PC2), thereby confirming the absence of cross-correlation between periods of high and low water. In addition, the findings indicate that the mid water season overlaps with both the high and low water seasons, according to PC1 and PC2 (Fig. 7a).

PCA revealed a some similarity between the high and mid water regime, partly influenced by anthropogenic factors, reflected in elements such as Ti, Zn, Cu, and Pb, which have been previously identified as indicators for industry and urban and agricultural emissions and runoffs (Zhang et al. 2009). Further analysis reveals the presence of shared factors (elements) between high and mid water regimes. These include (natural) Al in combination with B, while Se can be accumulated in domestic wastewaters, e.g., from coal combustion (Guinoiseau et al. 2018; Tian et al. 2010). Seasonal variations are explained by PC2 (Fig. 7a). Samples collected during the low water regime show lower component values for PC2, whereas samples collected during the mid and high water regimes generally exhibit higher PC2 values. The PCA also showed several groups of elements (Fig. 7b). The first group of elements is characterized by a high component loading on PC1. These are mainly alkali metals and alkaline earth elements, which form relatively mobile cations: K, Ca, Mg, Na, Rb, and Ba. B, which belongs to this group, also forms water soluble cations, while Mo is a metal that forms less soluble cations but might originate from similar geological formations as soluble cations. Weathering of feldspar, plagioclases, hornblende, and mica minerals might explain this group of elements. The second group of elements is characterized by a high component loading on PC2: Se, Al, Ni, Fe, Ti, and V. All of these elements often form immobile oxides and might represent less mobile fractions. Weathering of heavy mineral fractions can explain this group of elements. The characteristic of the third group is the negative component load in the PC2. These elements are U, Sr, Mn, Ca, and Mg. This group might show carbonate weathering as the third controlling factor of water chemistry, also explaining the main origin of ^87^Sr/^86^Sr in the Mur River system. PCA also revealed that the water regime might control the mobility of less soluble fractions, which are not mobile in the low water regime but more mobile in the mid and the high water regime. The fourth group of analytes (As, Cu, Zn, Pb, Cr, Cd, and ^87^Sr/^86^Sr) does not show any distinctive loading on any of the principal components. This suggests that their mobility is not controlled by the water regime in the same way as for other analytes with high absolute loadings on PC2. Instead, these elements appear to have independent sources, separate from the three previously discussed controlling factors. This may be explained by anthropogenic influences (e.g., Cu, Zn, Pb, and Cd), by natural sources independent of the geological background (e.g., Sr isotope ratio, As), or by a combination of both.

Wildi et al. (2004) conducted a study on sediments in Alpine rivers, lakes, and reservoirs downstream of urban areas in Switzerland. The study concluded that groundwater flows can potentially release heavy metals captured in sediments into the water phase. This may also be applicable for the Mur River, as documented by Retter et al. (2021). Furthermore, precipitation events and thunderstorms can trigger the release of heavy metals and reactivate pollution of rivers from historic industry, as evidenced in the Mur River in the 1960 s (Kammel and Mebert 2011; Wildi et al. 2004). In this study, the mass concentrations of heavy metals (e.g., Zn, Cu, Pb, Cr, and Ni) in rivers were observed to be elevated during the spring and summer months, compared with the mass concentrations in February 2023. During the sampling campaigns conducted in May 2022 (high water regime), precipitation events occurred, while the area was affected by heavy thunderstorms prior to sampling in August 2022 (mid water regime), both resulting in elevated discharges from the surrounding area into the rivers. It should be noted that the immediate vicinity of the Mur River is characterized by (former) industrial steel plants and paper factories. During the low water regime, water was captured in ice and snow, resulting in reduced runoff and a mass fraction signature indicative of geological impact. It is important to note that mass concentrations of heavy metals in the Mur River and its tributaries did not exceed the limit values according to the Austrian guidelines for drinking water at any time (Trinkwasserverordnung, 2001).

As a consequence of heavy rainfall in June 2022, which led to a significantly higher discharge, the Sr mass concentrations measured in the samples of the main source and 10 m below the source, respectively, are significantly lower (*γ* = 11.0 ± 0.9 µg L^−1^ and *γ* = 44.4 ± 3.5 µg L^−1^ at the reference point, R2) than in August 2023 (*γ* = 19.2 ± 1.0 µg L^−1^ and *γ* = 121.3 ± 7.2 µg L^−1^ at the reference sampling point, R2; see Chapter "Spatio-temporal variability of ^87^Sr/^86^Sr isotope ratios and Sr mass concentrations in the Mur River catchment"). The isotope composition was not affected by the precipitation event, which could suggest the fact that the main source is supplied by groundwater during periods of flooding, and thus exhibits a consistent isotope composition over time, or that additional water sources do not carry a significant amount of Sr (Aubert et al. 2002).

Section 4 was designated as such owing to the lengthening of the river and the consequent increase in water discharge, which prevents small- and mid-sized tributaries from affecting the isotope pattern, hence no distinct changes in the temporal variation were monitored. The only significant influence was observed in the Mur River in Mellach (R31), where a difference between seasons is incontestable with ^87^Sr/^86^Sr isotope ratios ranging from 0.71088 to 0.71225. Owing to accessibility reasons, the location for this sampling was selected in close proximity to the tributary Kainach, which has a diverse catchment area, and is briefly joined by the Mur River before the sampling point. It is highly plausible that the Sr composition assigned to the Mur River main channel in Mellach represents the Sr composition of the Kainach River owing to incomplete intermixing after the mouth of the tributary. Even though the Sr isotope composition of the Kainach is not available, the Sulm, which orginates from a similar catchment area, also exhibits a higher Sr isotope signal.

### ^87^Sr/^86^Sr isotopes as a river monitoring and management tool

#### Tracing hydrogeochemical fluxes and anthropogenic impacts

In the transition zone (section 3, R16, St. Georgen ob Judenburg to sample R19; Fig. 2a), seasonal variations were observed, where the low water regime revealed a higher ^87^Sr/^86^Sr isotope ratio (i.e., St. Lorenzen/Knittelfeld, R19, ^87^Sr/^86^Sr = 0.71141 ± 0.00014) than the highwater regime (^87^Sr/^86^Sr = 0.71123 ± 0.00019) and the midwater regime (^87^Sr/^86^Sr = 0.71105 ± 0.00017). It is important to acknowledge the possible impact of tributaries with varying geological catchments and ^87^Sr/^86^Sr isotope compositions (Fig. 5). However, the water discharges of the Mur River and its associated tributaries in that area, including Pölsbach (T10) and Granitzenbach (T11), exhibit seasonal variability, although over seasons the water discharges of the creek Pölsbach (13.9 m^3^ s^−1^ in May 2022, 7.1 m^3^ s^−1^ in August 2022, and 2.8 m^3^ s^−1^ in February 2023) and of the Mur in Zeltweg (Table 1) show a consistent ratio of 1:7. Moreover, the relative differences between June 2022 and November 2023 expressed as *Δ*-values for ^87^Sr/^86^Sr isotope ratios for the tributary Pölsbach in this area show values of 0.11‰. The lack of influence of the tributaries could indicate (1) an influence of rainwater (Pearce et al. 2015), of which the isotope signature is significantly lower in comparison to that of the river, and (2) a potential influence of hydrological reservoirs in high water settings, which balances the Sr isotope signatures (Aubert et al. 2002). In Retter et al. 2021 it could be demonstrated, that groundwater has a severe impact on the Mur River’s water body. The study comprehensively investigated *δ*^18^O isotope ratios to prove the impact of groundwater on the Mur River.

#### A region with history: traces of anthropogenic influences on the Mur River and its tributaries

The town of Leoben is the second largest city within the Mur River catchment area. Its topographical location in a mountainous region has historically contributed to its development as a center for the mining and steel industries. Until the 1980 s, industries were located in the vicinity of the tributaries, using them for the disposal of waste and effluent. The implementation of environmental protection legislation in Austria, and subsequently the European Union, has led to a significant enhancement in environmental awareness. Consequently, the rivers and creeks in the region (R20, St. Michael to R24, Bruck/Mur) are now characterized by their clarity and the absence of any indication of pollution. Although active pollution has ceased, industrial operations remain in the area close to several tributaries, including the Sonngrabenbach (T12), Lobmingbach (T13), Liesing (T14), Gößbach (T15), and Vordernberger Bach (T16) creeks.

The limited number of sampling sites within the Leoben area did not allow for statistically reliable interpretation; consequently, statistical analyses were omitted, and the elemental data were instead examined directly to explore potential trends and correlations. The results show distinct patterns of association among the samples: seasonal groupings corresponding to mid- and low-water conditions largely overlapped with each other and with the distribution of tributary samples. An anticorrelation was observed between Mg, Al, and Cu with the ^87^Sr/^86^Sr ratio. The anthropogenic trace elements Cu, Ni, Al, Mg, As, and Ti exhibited similar trends. The negative relationship of Cu and Fe with ^87^Sr/^86^Sr suggests that the Sr isotopic signature is predominantly geological in origin rather than influenced by anthropogenic input. Overall, three distinct elemental groupings can be distinguished: (1) soluble elements such as Ba, Sr, K, B, Na, and Ca; (2) elements forming insoluble oxides, including Ti, Fe, and V; and (3) a mixed group represented by Mg and Ni, which may indicate anthropogenic contributions, particularly considering the proximity of several sampling sites to the urban area. Moreover, in the case of the Mur River, low discharges from its tributaries did not significantly affect the main river’s geochemical signature.

#### Source assignment of river discharges by isotope pattern deconvolution (IPD) applied to the major tributary Mürz River

A significant shift in the ^87^Sr/^86^Sr isotope composition in the Mur River was observed during mid and low water regimes and between sampling points Unteraich (R23) and Bruck an der Mur (R24), which are only 3 km apart. This revealed how the Mürz River (T17, Fig. 5b) contributes to the isotope composition of the Mur River. The results obtained by IPD indicate that the Mürz, as one of the major tributaries to the Mur, has a significantly different Sr isotope composition (see sections “Spatial variations along the Mur River and tributary contributions” and “Influence of tributaries in sections 3 and 4, south of Bruck/Mur”). This difference leads to a change in the Sr isotope composition of the main river, with an estimated contribution of 17% from the Mürz, whereas 83% of the Sr isotope signature after the confluence can be attributed to the main river (ESI-2.8). This alteration affects the Sr isotope ratios observed in Bruck an der Mur, which is also in line taking into account the water discharges of Mur River and Mürz (Table 1 and 8.26 m^3^ s^−1^, respectively) and the Sr mass concentrations (*γ* = 203.2 ± 20.3 µg L^−1^ and *γ* = 187.8 ± 18.8 µg L^−1^, respectively) of both rivers. The Mürz tributary has been demonstrated to have a significant influence on the Mur River, with the potential to overwrite the signature of the isotope composition of the river’s underlying bedrock (Zieliński et al. 2016).

#### Isotope insights for sustainable Alpine management

Environmental monitoring in Alpine regions is essential for understanding ecological dynamics and informing resource management practices. The investigation of Sr isotopes in Alpine rivers provides valuable insights into geological influences, hydrological processes, and biological interactions. This aspect is particularly crucial in the context of a developing valley such as the Mur Valley. A recent project, for example, has been initiated with the aim of reactivating ancient deposits rich in valuable ores, including Au, Ag, Cu, Fe, As, and other critical raw materials (Dunkel et al. 2025). The reactivation of these deposits is expected to potentially influence the Mur River system through various tributaries. The Sr isotope ratio basemap established in this study provides a valuable tool for detecting and assessing such anthropogenic impacts. It enables evaluation of whether these activities exert a significant influence on the river system and whether alterations are reflected primarily in the water masses, the sediment load, or the overall catchment isotope signature. Similar studies in other river catchments have shown similar findings. A study on Sr isotopes in the Austrian Alpine Foreland, for example, revealed how variations in isotope signatures can indicate shifts in water sources and catchment geology, which are crucial for managing freshwater resources and assessing ecosystem health (Zitek et al. 2023). In addition, isotope analyses contribute to broader ecological studies by linking isotope data to species movement and habitat utilization, thereby enhancing our understanding of biodiversity and ecosystem function in these sensitive environments (Hobson 2008). As an example for such movements, the ^87^Sr/^86^Sr isotope composition and Sr/Ca ratios of fish otoliths can be utilized (Avigliano et al. 2021; Shao et al. 2018; Zitek et al. 2023). The Mur River, like other Alpine rivers, is subject to strong regulation by dams, which on one hand is important for the production of energy but on the other hand has the effect of reducing the fish population (Pinter et al. 2024; Verbund 2024; Curtean-Bănăduc et al. 2019). This is particularly relevant given the impending construction of new dams. In order to ascertain the effects of such barriers on fish migration, (aquatic) isoscapes can be used as an indicator for long-distance fish migration and for determining their origin and provenance (Zitek et al. 2015). By integrating isotope monitoring into resource management strategies, stakeholders can make informed decisions that promote sustainable practices in Alpine regions and beyond (Zitek and Schmutz 2012). Sr isotopes can thus be used as measure for river connectivity and further as a measure for the assessment of risks to an ecosystem and its biodiversity.

## Conclusions

The findings of this study contribute significantly to the understanding of strontium isotope dynamics in Alpine river systems, offering valuable perspectives on the stability of isotope signatures and the factors influencing them. The established base for an isoscape provides a robust framework for tracing geological inputs and possible anthropogenic impacts on freshwater systems, with broad implications for environmental monitoring, ecological studies, and resource management in Alpine regions and beyond. The Mur River embodies many of the challenges faced by European rivers today, including climate stress, industrial impacts, hydropower management, and biodiversity loss. This dataset, obtained from studying the Mur River at a rather small scale, can help to understand other river systems, aiding in the development of holistic management strategies that balance human needs with ecological sustainability. This is especially crucial as Europe faces increasing environmental stressors such as drought, extreme weather, and rising energy demands.

Notably, the distinct variations in isotope ratios across different sections of the river reflect the complex interplay between geological formations and hydrological influences, particularly from tributaries. The findings indicate that most sections of the river align with expected geological classifications. The seasonal fluctuations observed highlight the impact of precipitation and groundwater interactions on isotope composition, underlining the necessity for continuous environmental monitoring. Furthermore, the results highlight the resilience of isotope signals in the main river despite short-term disturbances from tributary inflows during extreme events, offering insights into long-term river monitoring and management.

Thus, the comprehensive dataset obtained provides a solid foundation for further in-depth investigations aimed at characterizing the catchment area and proves the versatility of strontium isotope ratios as environmental indicator. It also enables the assessment of potential anthropogenic influences and supports evaluations relevant to freshwater fauna migration—an essential aspect for informing effective river management strategies. Beyond these immediate applications, the approach highlights the potential of integrating isotope analysis into broader monitoring frameworks, offering valuable spatial and temporal insights into element sources and ecological processes. By bridging the gap between research and application, this work contributes to the development of more sustainable riverine ecosystem management in the face of growing environmental pressures.

## Supplementary Information

Below is the link to the electronic supplementary material.

Supplementary file1 (PDF 467 KB)

Supplementary file2 (XLSX 361 KB)

## Data Availability

No datasets were generated or analyzed during the current study.

## Declarations

### Generative AI and AI-assisted technologies in the writing process

In preparation of this work, the authors used the generative AI technology DeepL write (DeepL SE, Germany) and Chat GPT 4.o [OpenAI. (2023)] in order to improve language and readability. After using this tool, the authors reviewed and edited the content as necessary and take full responsibility for the content of the publication.
