# Supplementary material for: Spatio-temporal variations of strontium isotope ratios in the Mur River: a tool to support river management
Source: Aquat Sci. 2026 Jan 17;88(2):25. doi: 10.1007/s00027-025-01253-4 (PMC12811288; doi:10.1007/s00027-025-01253-4)
Supplement: Supplementary file 1 — Supplementary file1 (PDF 467 KB) [file 27_2025_1253_MOESM1_ESM.pdf]

Supplementary Information – ESI-1

Spatio-Temporal Variations of Strontium Isotope Ratios in the Mur River: A Tool to Support River Management

Ulrike Moser[1], Barbara Čeplak[2], Stefan Wagner[1], Shaun T. Lancaster[1], Martin Šala[3], Thomas Prohaska[1,4], Gorazd Žibret[2], Johanna Irrgeher[1,4,*]

[1] Montanuniversität Leoben, Department General, Analytical and Physical Chemistry, Chair of General and Analytical Chemistry, Austria

[2] Geological Survey of Slovenia, Dimičeva ulica 14, 1000 Ljubljana, Slovenia

[3] National Institute of Chemistry, Hajdrihova ulica 19, 1000 Ljubljana, Slovenia

[4] University of Calgary, Department of Physics and Astronomy, T2N 1N4 Calgary, Canada

*Correspondence: johanna.irrgeher@unileoben.ac.at

ESI-1.1 Calibration standards and quality control solutions for ICP-MS(/MS)

The ICP multi-element calibration standard stock solution VI (Merck Certipur, Darmstadt, Germany), herein referred to as "MVI", contains 989 mg L^{-1} of Ca; 10 mg L^{-1} of Sr; and 9.9 mg L^{-1} Rb. By diluting with nitric acid (HNO_3 w = 2 %), a 12-point calibration was generated ranging from 0.005 to 75.0 ng g^{-1} Sr.

Traceability of MVI is given according to NIST Standard Reference Materials and can be found in Table ESI-1.T1.

ESI-1.T1 Traceability of MVI according to NIST

Element	NIST Standard Reference Material
**Ca**	SRM 3109a
**Rb**	SRM 3145a
**Sr**	SRM 3153a

For quality control (QC), standards were prepared in order to check the calibration and possible drift effects. A mixture of single-element standard solutions of Ca 1000 mg L^{-1} (Merck KGaA, Darmstadt, Germany) and Rb 1000 mg L^{-1} (Merck KGaA, Darmstadt, Germany) was spiked to a MVI calibration standard. Mass fractions after dilution with HNO_3 (w = 2 %) can be found in Table ESI-1.T2.

ESI-1.T2 Mass fractions of QC standards for ICP-MS

Analyte	Mass fraction [ng g^{-1}]
**Rb, Sr**	24
**Ca**	938

ESI-1.2 Instrument Parameters

ESI-1.T3 Instrumental parameters for quantitative measurements at Montanuniversität Leoben (MUL) and the National Institute for Chemistry in Slovenia (NIC).

	MUL	MUL	NIC	NIC
**Parameter**	**PerkinElmer NexION 5000 ICP-MS/MS**	**PerkinElmer NexION 2000 ICP-MS**	**Agilent 7850 ICP-MS**	**Varian 715-ES ICP Optical Emission Spectrometer**
**Analytes**	Ca, Rb, Sr	Ca, Rb, Sr	B, Al, Ti, V, Cr, Mn, Fe, Ni, Cu, Zn, As, Se, Rb, Sr, Mo, Cd, Ba, Pb, U	Na, Mg, K, Ca
**Spray chamber temperature**	5 °C	5 °C	2 °C	2°C to 5°C
**Interface cones**	Nickel	Nickel	Nickel	Nickel
**Nebulizer**	PFA-ST-40 44296	PFA ST3-40 46387	MicroMist	glass concentric nebulizer
**Nebulizer gas flow**	0.97-0.99 mL min^{-1}	0.93-0.99 mL min^{-1}	1.05 L/min	
**RF power**	1600 W	1600 W	1550 W	1.2 kW to 1.5 kW
**Plasma gas flow**	16 L min^{-1}	16 L min^{-1}	15 L min^{-1}	15 L min^{-1}
**Auxiliary gas flow**	1.3 L min^{-1}	1.4 L min^{-1}	1.0 L min^{-1}	1.0 L min^{-1}
**Data acquisition mode**	6 sweeps/reading, 1 reading/replicate, 6 replicates	6 sweeps/reading, 1 reading/replicate, 6 replicates	10 sweeps/replicate, 3 replicates	10 sweeps/replicate, 3 replicates
**Dwell time per replicate**	25-150 ms	50 ms	50 - 1000 ms	
**Integration time**	150-900 ms	300 ms	0.1 - 3.0 Integ Time/Mass (sec)	
**RPa**	0-0.02 V	0 V		
**RPq**	0.25 V	0.25 V		

ESI-1.T4 Instrumental parameters for MC-ICP-MS

Parameter	NU Plasma HR	
**RF Power**	1300	
**Coolant flow**	13 L/min	
**Auxiliary gas flow**	0.9 L/min	
**Nebulizer pressure**	29-34.4 psi	
**Interface cones**	Nickel, dry	
**Nebulizer**	Aridus II (Teledyne CETAC) with MicroFlow PFA-ST nebulizer (ESI)	
**Measurement mode**	Static batch analysis (6 blocks with 10 measurement cycles with 10 s integration time)	
**Resolution mode**	m/Δ = ~300	
**Cup configuration**	H6: ^{91}Zr; H5: ^{90}Zr; H2: ^{88}Sr; Ax: ^{87}Sr; L2: ^{86}Sr; L3: ^{85}Rb; L4: ^{84}Sr	
**Axial mass / mass separation**	87/0.5	
**Average sensitivity**	50 ng g^{-1} Sr	20 ng g^{-1} Sr
	109-207 V/(µg g^{-1})	122 V/(µg g^{-1})

ESI-1.3 Sr/Ca elemental mass concentration ratio

Sr mass concentrations were normalised to Ca mass concentrations and multiplied by 1000 in order to compare the Sr mass concentrations throughout the seasons, and thus a potential impact of the tributaries on the river could be evaluated (Supplementary Figure 1). The $\gamma(Sr)/\gamma(Ca) \times 10^3$, further referred to as Sr/Ca $\times 10^3$ elemental ratios in the Mur River ranged from 3.42 (R25, Mixnitz) to 5.52 (R11, Predlitz) in May 2022, from 4.12 (R17, Niklasdorf) to 9.52 (R13, St. Lorenzen/Mur) in August 2022 and from 3.38 (R31, Mellach) to 9.06 (R8, St. Michael/Lungau) in February 2023. The lowest Sr/Ca $\times 10^3$ ratios were observed during high water regime in May 2022, while the highest ratios were recorded during the mid-water regime in August 2022. Despite the similarity in pattern across the three seasons, there were notable variations observed, particularly during the mid and low water regimes. Peaks were identified during the mid and low water season in St. Michael/Lungau (R8) and in St. Lorenzen/Mur (R13) during the mid-water regime. The principal differences were identified in section 1 (Fig. 2), in section 2 the Sr/Ca $\times 10^3$ ratios decreased gradually until they reached a level in section 3, with exceptions for single peaks and drops (e.g., Sladki vrh, R35, August 2023; Mellach, R31, February 2023). In the tributaries, the highest Sr/Ca $\times 10^3$ ratio was identified in the Lorenzer Bach (T7) with a value of 8.5 recorded in February 2023. In contrast, the lowest ratio was observed in Rötschbach (T20) with a value of 1.82 recorded in November 2023. The fluctuations in Sr mass concentration (Supplementary Fig. 1) at the respective points often correspond to those in and decreases in the Sr/Ca $\times 10^3$ elemental mass concentration ratios.

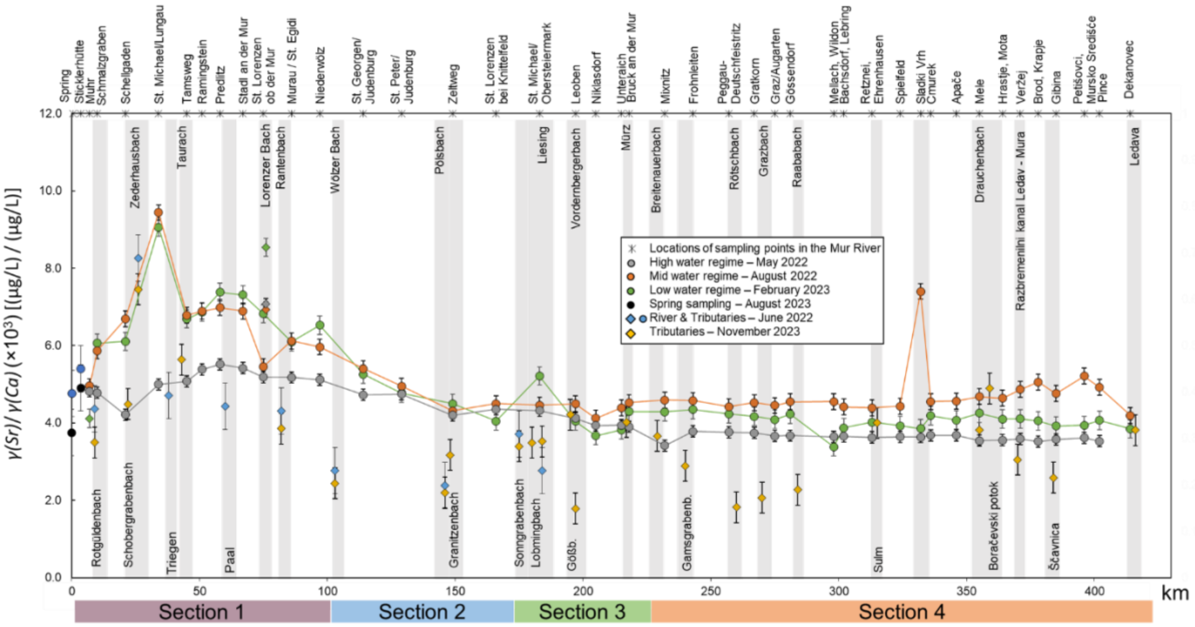

Supplementary Fig. 1 γ(Sr) normalized to γ(Ca) and multiplied times 1000. $\gamma(Sr)/\gamma(Ca)$ = (µg L^{-1}) / (µg L^{-1}). The position of the tributary labels indicates if they tribute on the left side of the river in flow direction (top) or from the right side (bottom). Connecting lines between datapoints represent the course of the Mur River. Error bars show expanded uncertainty *U* representing 2 SD of replicate analysis of three individual samples.

ESI-1.4 Analysis of triplicate samples from May 2022

The results of $^{87}Sr/^{86}Sr$ isotope ratio analysis of the water samples, conducted in triplicate samples of one location generally show high homogeneity of the samples reflected in a small relative variation between replicate analysis. The delta (*Δ*) values across the three replicates varied from -0.41 ‰ to 0.38 ‰ (see Table ESI-2.8). The average *Δ* (-0.2 ‰ to 0.06 ‰) of all measurements lies within the range of a single standard deviation of a measurement. In consequence, one single replicate per sample site can be considered as representative for a location and thus also resources can be saved both for economic and environmental reasons.
